# The travelling particles: community dynamics of biofilms on microplastics transferred along a salinity gradient

**DOI:** 10.1038/s43705-022-00117-4

**Published:** 2022-04-11

**Authors:** Jessica Song, Lukas Beule, Elanor Jongmans-Hochschulz, Antje Wichels, Gunnar Gerdts

**Affiliations:** 1 Department of Microbial Ecology, Biologische Anstalt Helgoland, Alfred Wegener Institute Helmholtz Centre for Polar and Marine Research, 27498 Helgoland, Germany; 2 Julius Kühn Institute—Federal Research Centre for Cultivated Plants, Institute for Ecological Chemistry, Plant Analysis and Stored Product Protection, Königin-Luise-Strasse 19, 14195 Berlin, Germany

**Keywords:** Microbial ecology, Biofilms, Microbial ecology

## Abstract

Microplastics (MP), as novel substrata for microbial colonization within aquatic ecosystems, are a matter of growing concern due to their potential to propagate foreign or invasive species across different environments. MP are known to harbour a diversity of microorganisms, yet little is understood of the dynamics of their biofilms and their capacity to successfully displace these microorganisms across different aquatic ecosystems typically marked by steep salinity gradients. To address this, we performed an in situ sequential incubation experiment to simulate MP transport from riverine to coastal seawaters using synthetic (high-density polyethylene, HDPE and tyre wear, TW) and natural (Wood) substrata. Bacterial communities on incubated particles were compared to each other as well as to those in surrounding waters, and their dynamics along the gradient investigated. All communities differed significantly from each other in their overall structure along the salinity gradient and were shaped by different ecological processes. While HDPE communities were governed by environmental selection, those on TW and Wood were dominated by stochastic events of dispersal and drift. Upon transfer into coastal seawaters, an almost complete turnover was observed among HDPE and TW communities. While synthetic particles displaced a minor proportion of communities across the salinity gradient, some of these comprised putatively pathogenic and resistant taxa. Our findings present an extensive assessment of MP biofilms and their dynamics upon displacement across different aquatic systems, presenting new insights into the role of MP as transport vectors.

## Introduction

Since the advent of microplastic (MP) research, scientists have endeavoured to draw a clearer picture of the distribution and impact of these synthetic items throughout their largest sink—the ocean [1]. The introduction of MP into such dynamic ecosystems has birthed a myriad of concerns regarding the extent of their influence on surrounding environments [2, 3]. Due to their persistent and highly dispersible nature, one such concern is the capacity of MP to harbour and propagate microbial assemblages across different ecosystems, potentially introducing harmful or invasive species into foreign environments [4]. Upon entering the environment, the physicochemical properties of MP are rapidly altered, resulting in the accumulation of an organic-rich matrix on the surfaces of these substrata. These conditioning films offer microorganisms better access to nutritional resources and in hydrodynamic environments additionally provide a foundation for greater stability and enhanced community interactions [5, 6]. As such, MP have been demonstrated to harbour a wide diversity of microorganisms across a range of aquatic environments [7–9], some of which include potentially harmful bacteria [10, 11].

MP have been posited through several studies to act as a novel niche within aquatic environments, with MP-associated communities found to be distinct from those in surrounding waters [12–14]. Additionally, evidence of niche partitioning between different polymer types was reported by Frère et al. [15], where polystyrene particles were observed to harbour communities discrete from other polymer types. Substrate-specificity, however, remains a matter heavily contended as other studies have suggested that surrounding environmental conditions take precedence over substrate type in shaping biofilm communities [8, 13]. To test this hypothesis, Amaral-Zettler et al. [16] compared MP sampled from different oceanic gyres and found that patterns observed among biofilm communities were more reflective of their surrounding environment than substrate preference. Their investigations later extended to Mediterranean basins, where the potential of MP to displace communities from rivers into open seawaters were additionally assessed [17]. In their study, a small overlap was observed when comparing MP biofilms sampled from rivers and ports to those from marine waters. Conclusive deductions, however, have yet to be made on the actual connectivity of these contrasting environments. Among the different abiotic conditions that have been found to play a role in shaping MP biofilms, salinity has been postulated as one of the greatest governing factors [8, 18, 19], further challenging the notion of a MP-mediated displacement of microbial communities across different aquatic environments.

While the threat of a MP-mediated propagation of microorganisms throughout aquatic ecosystems remains a central concern, still very little is understood of the processes that drive the formation of these biofilms and the capacity of MP to successfully displace these communities across the strong salinity gradients that mark these different systems [20]. As rivers have been identified as a major pathway for the entry of MP into global oceans [21], it has become particularly important to better understand the dynamics and transport of microbial communities on MP along this particular axis. The primary objective of our study was to investigate the effect of a salinity gradient on the dynamics of MP-associated bacterial communities upon their transport from freshwater to marine environments. We studied firstly the diversity and structure of communities on the surfaces of synthetic particles sequentially incubated along a transect that spanned from riverine to coastal seawater conditions and compared them to those found on natural substrata as well as surrounding waterborne communities. The turnover and assembly of each of the different communities upon transfer along the gradient were analysed to assess their dynamics as well as their potential displacement across different aquatic environments.

## Materials and methods

### Sample incubation and collection

**Fig. 1:**
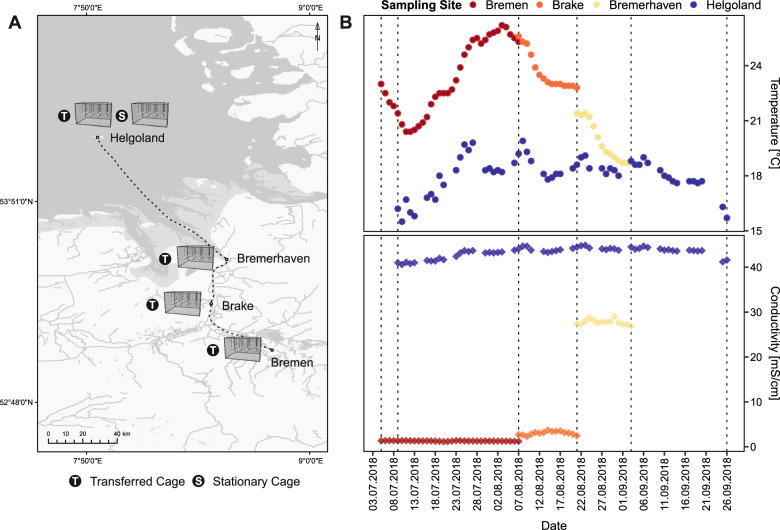
The sequential incubation experiment was performed along a salinity gradient, ranging from freshwater to coastal seawater conditions. **A** Map of the four incubation sites (Bremen, Brake, Bremerhaven, Helgoland) spanning the Weser River to the offshore island of Helgoland (North Sea) in Germany. Two cages were set up; the first (Transferred Cage) was sequentially incubated along the salinity gradient while the second (Stationary Cage) remained in the offshore site throughout the entire duration of the experiment, acting as a marine control. **B** Daily conductivity and temperature at each site throughout each respective incubation period. Vertical dashed lines are indicative of sampling dates. Source: *Senator für Umwelt, Bau und Verkehr (Bremen); Niedersächsischer Landesbetrieb für Wasserwirtschaft, Küsten- und Naturschutz, NLWKN (Brake); Wasserstraßen- und Schifffahrtsamt Bremerhaven (Bremerhaven); Biologische Anstalt Helgoland, Alfred-Wegener-Institut (Helgoland)*.

A sequential incubation experiment was conducted from July to September 2018 along a salinity gradient that spanned the transect from the Weser River to the offshore island of Helgoland (North Sea) in Germany (Fig. 1a).

Synthetic particles of approximately 4 mm, consisting of high-density polyethylene (HDPE, ExxonMobil™ HDPE HTA 108, Irving, TX, USA) and tyre wear (TW, Genan Coarse, Genan GmbH, Dorsten, Germany), were enclosed within stainless steel mesh capsules along with natural wood particles of similar diameters (Hobbyshop Rüther GmbH & Co., Berlin, Germany), and contained within two metal cages (60 × 40 × 30 cm, Fischer-Draht GmbH, Winterlingen, Germany). One cage (Transfer), comprising 36 capsules (12 × HDPE, 12 × TW, 12 × Wood), was sequentially incubated along four sites (Bremen, Brake, Bremerhaven, Helgoland), for predetermined periods of time reflective of modelled retention times at each site. Particles were first exposed to freshwater conditions (daily mean conductivity = 1.16–1.37 mS/cm) upstream of the weir in Bremen for 33 days, with no tidal influence due to the presence of the weir. The cage was then transferred to Brake, located roughly 40 km from the river mouth, where particles were incubated for 14 days. These waters, comprising the tidal freshwater reaches of the estuary, generally receive a well-mixed influence of marine and freshwater (daily mean conductivity = 2.32–3.72) and are characterized by oligohaline conditions. The third site was situated by the river mouth in Bremerhaven and marks the border of the reach. In these brackish waters, particles were exposed to strong salinity shifts due to incoming tides (daily mean conductivity = 26.93–29.03 mS/cm) over a period of 13 days. Lastly, at the final site located off the island of Helgoland, particles were incubated for 23 days in coastal seawaters characterized by highly saline conditions (daily mean conductivity = 41.20–44.67 mS/cm) and year-round mixing. Concurrently, the other cage (Stationary), comprising 9 capsules (3 × HDPE, 3 × TW, 3 × Wood), was incubated in the final offshore site (Helgoland) throughout the entire duration of the experiment as a marine control.

For the transferred cage, three capsules of each type were collected following incubation at each site and surrounding waters were sampled at the beginning and end of each incubation period to provide a comparison against source communities. Abiotic conditions were similarly measured before and after each incubation using a multi-probe (Multi3430, WTW, Weilheim, Germany). Additionally, data on the conductivity and temperature at each site throughout the incubation experiment were obtained from the responsible agencies and displayed in Fig. 1b. Following each sampling event, the cage was transferred to the subsequent site for incubation and the entire sampling procedure repeated. More detailed descriptions of the experimental setup are provided by Song et al. [22] and in the supplement.

### Sample preparation and amplicon sequencing

Total biofilm DNA was extracted from incubated particles using the DNeasy PowerBiofilm kit (QIAGEN, Hilden, Germany) according to the manufacturer’s instructions. Waterborne communities were analysed by serial filtration through sterile 3.0 µm and 0.2 µm polycarbonate filters (47 mm, Merck Millipore, Darmstadt, Germany) to assess both the particle-associated (Water 3, > 3 µm) and free-living (Water 02, 0.2–3 µm) fractions, respectively. DNA of waterborne communities were isolated using the DNeasy PowerWater kit (QIAGEN, Hilden, Germany) as per the manufacturer’s instructions. Details on starting sample material and DNA concentrations are provided in Table S1.

Library preparation and amplicon sequencing were performed by LGC Genomics GmbH (Berlin, Germany). Bacterial 16S rRNA genes were amplified using the primer pair 341F (5′-CCTACGGGNGGCWGCAG-3′) and 785R (5′-GACTACHVGGGTATCTAAKCC-3′) [23], targeting the V3-V4 hypervariable regions. 16S amplicons were then subject to sequencing using an Illumina MiSeq (2 × 300 bp, V3 chemistry) and adaptors and primers removed from the resulting reads.

### Read processing

Reads were processed on the QIIME 2™ platform (v.2020.8 – 2021.2) [24]. Quality control was performed on demultiplexed paired-end reads using the DADA2 (Divisive Amplicon Denoising Algorithm) plugin [25]. Amplicon sequence variants (ASVs) inferred were filtered to remove singletons and subject to taxonomic classification using a multinomial naive Bayes classifier [26] trained on full-length 16S rRNA sequences from the SILVA v132 database [27]. Non-bacterial reads were removed from the dataset. A phylogenetic insertion tree was constructed using a SATé-enabled phylogenetic placement technique [28] based on the SILVA v128 reference database.

### Diversity analyses

Analyses of processed reads were performed in R (v.4.0.3 onwards) [29]. Reads were normalized by scaling with ranked subsampling using the *SRS* package [30] and a species richness curve generated based on the Shannon diversity index to ensure a sufficient sampling depth at the normalized library count. For downstream analyses, reads from waterborne communities sampled at the start and end of each incubation period were pooled to represent a single timepoint and more comprehensively assess waterborne communities throughout each incubation period.

Both the taxonomic and phylogenetic diversity of communities were investigated in this study. Shannon and Gini-Simpson indices were calculated using the *vegan* package [31] to estimate species richness. while the diversity of bacterial lineages and their degree of relatedness were measured based on Faith’s PD and nearest taxon index (NTI), respectively, using the *picante* package [32]. The standardized effect size of Faith’s PD (ses.PD) was additionally calculated to determine whether observed diversity was greater or lower than expected by chance. Any significant differences in diversity between sample types were determined through a Kruskal–Wallis Rank Sums test and post-hoc Dunn’s test using the *stats* and *FSA* packages [33].

### Compositional analyses

Significant compositional differences between sample types were explored through differential abundance testing using the *metacoder* package [34]. To elucidate changes within each sample type upon transfer along the salinity gradient, a similarity percentage analysis was performed in PRIMER v7 [35], where the taxa responsible for driving observed changes and their relative contributions were determined. Additionally, ASV-level differences between sample types were visualized using the *UpSetR* package [36] to assess the number of ASVs shared between sample types and the proportion of ASVs successively displaced along the gradient.

### Community structure and turnover

Dissimilarities in the taxonomic and phylogenetic structure between and within sample types were investigated. Using the *vegan* package, weighted and unweighted taxonomic dissimilarities were computed based on Bray–Curtis and Jaccard indices, respectively, while weighted and unweighted UniFrac distances were calculated using the *phyloseq* package [37] to assess phylogenetic dissimilarities. Structural variations were partitioned using a permutational multivariate analysis of variance (PERMANOVA) [38] performed in PRIMER v7. A two-way factorial design was employed to test the effect of Sample Type and Sampling Site as explanatory variables on the observed dissimilarities, as well as their interaction term. Post-hoc tests were performed to further explore the levels of significant distinctions, with Sampling Site as a fixed factor to discriminate between sample types at each site and with Sample Type as a fixed factor to elucidate differences within each sample type across the four sites. Homogeneity of multivariate dispersions within groups was tested using PERMDISP. A one-way PERMANOVA and post-hoc tests were additionally conducted on samples collected from the final offshore site alone to determine any significant structural differences between transferred and stationary particle biofilms.

Dissimilarities were partitioned based on the Jaccard index into components of turnover and nestedness using the *betapart* package [39] to investigate whether variations between sites were the product of species replacement or species loss/gain. Patterns of taxonomic and phylogenetic dissimilarities were further explored through a principal coordinate analysis (PCoA) using the *ape* [40] and *phyloseq* packages, respectively [41].

### Community assembly

A null model framework [42] was implemented using the *picante* package and custom scripts [43, 44] to identify the processes that shape each community along the salinity gradient and their relative contributions. Mantel correlograms were first computed to test for a significant relationship between the most closely-related taxa and their inhabited ecological niches (phylogenetic signal). Following the detection of a significant signal, the compositional turnover of each community between sites was quantified through pairwise comparisons based on the ß-nearest taxon index (ßNTI) and compared against null models. Deviations from the null distribution are indicative of variable and homogenous selection (ßNTI > 2 and ßNTI < −2, respectively) and selection discounted as a dominant influence when no significant deviations are observed (−2 < ßNTI < 2). The relative contributions of the processes that govern these stochastically-assembled communities were then estimated using the Raup-Crick metric [45] based on Bray–Curtis dissimilarities (RC_bray_). Deviations from the null expectation indicate dispersal limitation (RC_bray_ > 0.95) or homogenizing dispersal (RC_bray_ < -0.95), while values that fall within the null distribution (−0.95 < RC_bray_ < 0.95) suggest a turnover undominated by selection or dispersal, otherwise referred to as ecological drift.

### Data repository and processing scripts

16S amplicon sequences generated in this study have been deposited in the European Nucleotide Archive at EMBL-EBI under accession number PRJEB47707. Processing scripts are made available at https://github.com/jessicaxsong/TheTravellingParticles.

## Results

### Bacterial community composition

**Fig. 2:**
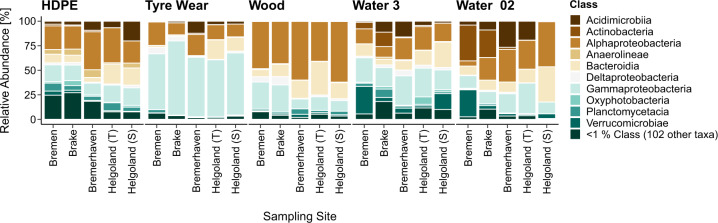
All sample types were dominated by three common bacterial classes. Relative proportions of bacterial classes detected for each sample type at each site. All classes with a mean relative abundance below 1% were grouped into one category (<1% Class). Helgoland (T) and (S) represent samples from the transferred and stationary cages, respectively. For particle-associated (Water 3) and free-living (Water 02) waterborne communities, Helgoland (T) and (S) refer to surface waters sampled from the final offshore site before and after the incubation of the transferred and stationary cages, respectively, which were pooled together.

Bacterial communities were profiled through 16S amplicon sequencing, from which 13 320 unique ASVs were inferred and classified into 8 phyla and 10 classes with a mean relative abundance greater than 1%. Overall communities detected for all sample types consisted predominantly of *Proteobacteria* (mean relative abundance > 40% each, Table S2), and were dominated by three common classes, *Alphaproteobacteria*, *Gammaproteobacteria*, and *Bacteroidia* (Fig. 2, Table S3). *Alphaproteobacteria* comprised the largest proportion of HDPE, Wood, and free-living waterborne communities (Water 02) while TW and particle-associated waterborne communities (Water 3) were predominated by *Gammaproteobacteria*. A significantly greater abundance of *Gammaproteobacteria* and *Alphaproteobacteria* were detected on TW and Wood, respectively, each constituting a mean relative abundance of more than 50% of their overall communities (Fig. S2, Table S3). TW-associated *Gammaproteobacteria* were composed mostly of *Oceanospirillales* (Fig. S3), with significantly greater counts of families such as *Pseudohongiellaceae*, *Oleiphilaceae*, and SS1-B-06-26. *Alphaproteobacteria* detected on Wood consisted mainly of *Rhodobacterales* and *Sphingomonadales*. Similarly, HDPE-associated *Alphaproteobacteria* were composed predominantly of *Rhodobacterales*. HDPE communities, however, were more heterogenous than those of other sample types and consisted of more low-abundance classes. Classes with a mean relative abundance of less than 1% collectively represented 8–27% of HDPE communities across sites. As such, differences between HDPE and other sample types were marked by less abundant phyla, such as *Chloroflexi* (6%) and *Cyanobacteria* (2%), along with others that made up less than 1%.

Only a minor proportion of the total ASVs detected on the incubated particles were also found in surrounding waters (Fig. S4). HDPE shared the greatest proportion of their total ASVs in common with waterborne communities (19% Water 3; 3% Water 02) relative to TW and Wood (14% and 3%; 15% and 3%, respectively). Among the three substrata, HDPE and TW shared the most ASVs in common, followed by HDPE and Wood. The majority of the ASVs detected on HDPE and Wood (60% each) were distinctive to the respective substrata, while only about 41% of total ASVs on TW were unique.

### Taxonomic and phylogenetic diversity and structure

**Fig. 3:**
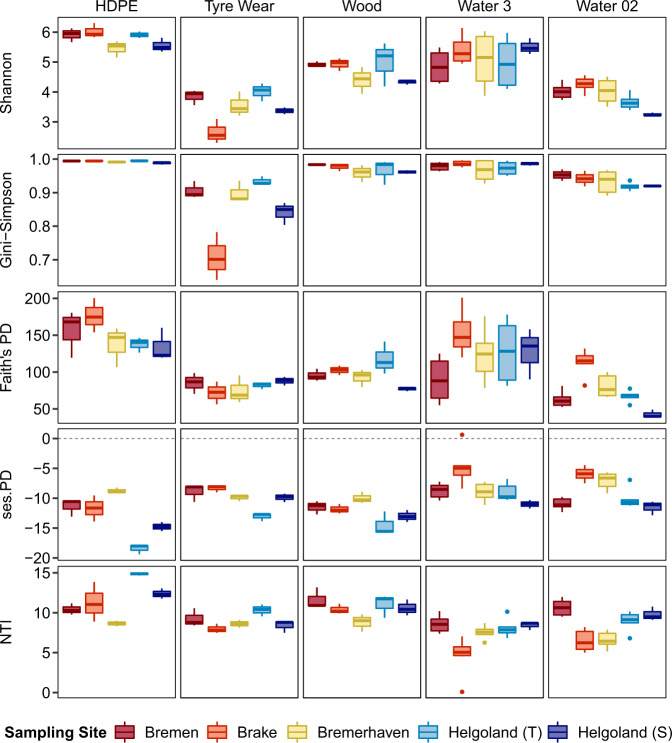
Taxonomic and phylogenetic diversity of the different communities at each site. Species richness of communities was calculated based on Shannon and Gini-Simpson indices, phylogenetic diversity based on Faith’s PD and its standardized effect size (ses.PD), and mean phylogenetic relatedness based on the nearest taxon index (NTI). Helgoland (T) and (S) represent samples from the transferred and stationary cages, respectively. For particle-associated (Water 3) and free-living (Water 02) waterborne communities, Helgoland (T) and (S) refer to surface waters sampled from the final offshore site before and after the incubation of the transferred and stationary cages, respectively, which were pooled together.

The taxonomic and phylogenetic diversity of HDPE communities were the highest of all sample types across all sites (Fig. 3). While significantly higher than other sample types, the overall diversity of HDPE communities was comparable to that of particle-associated waterborne communities (Tables S4–S7). TW communities, conversely, were the least diverse. The phylogenetic diversity of all communities was lower than expected by chance, as indicated by the negative ses.PD values computed. Additionally, positive NTI values were computed for all communities across sites, denoting phylogenetic clustering among the most closely-related taxa within each community. Communities detected on transferred particles did not differ significantly in their overall diversity from their stationary counterparts which remained in the offshore site throughout the duration of the experiment.

**Fig. 4:**
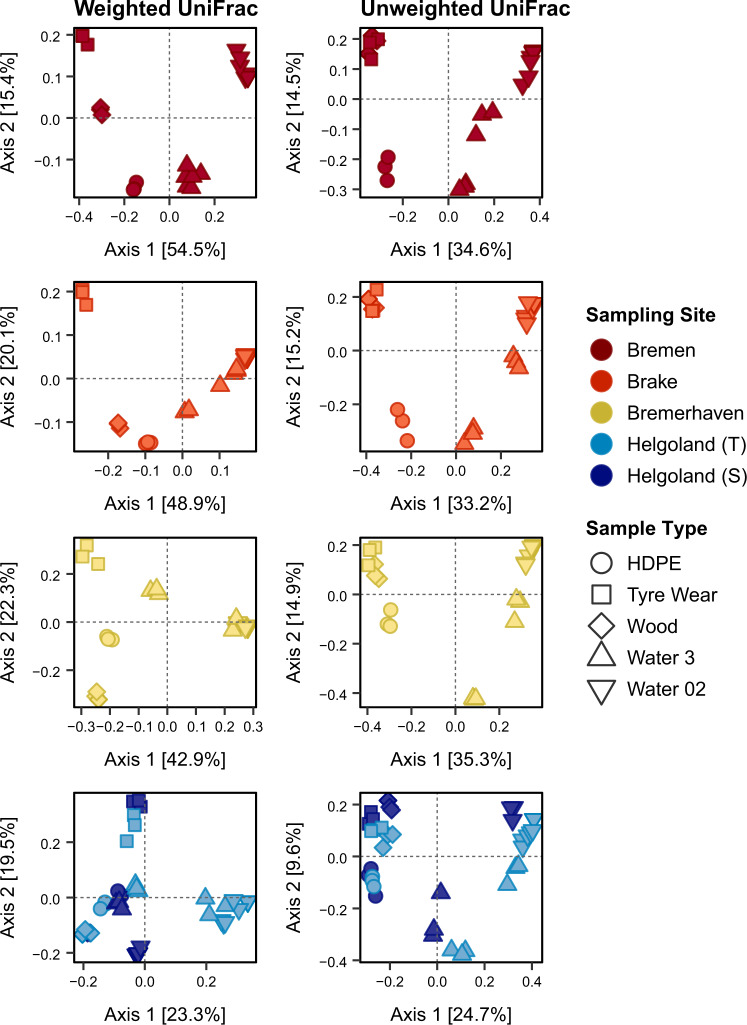
Communities of the different sample types were distinct in their phylogenetic structure at all sites. Phylogenetic dissimilarities were measured by weighted and unweighted UniFrac distances. Explained variation [%] along both Axes 1 and 2 are displayed in square brackets. Helgoland (T) and (S) represent samples from the transferred and stationary cages, respectively. For particle-associated (Water 3) and free-living (Water 02) waterborne communities, Helgoland (T) and (S) refer to surface waters sampled from the final offshore site before and after the incubation of the transferred and stationary cages, respectively, which were pooled together.

The effects of Sample Type and Sampling Site on structural dissimilarities were tested through PERMANOVA using a multifactorial design. Taxonomic and phylogenetic dissimilarities were shaped by both Sample Type and Sampling Site, with a significant interaction between both factors (Table 1). Overall community structure differed significantly between all sample types at each site (Table S8). Significant heterogeneity was observed within groups. This was largely due to the dispersion within particle-attached and free-living waterborne communities, which comprised pooled reads of each fraction sampled at the start and end of each incubation period that varied noticeably within groups. However, comparable trends of dissimilarity were observed in ordination space (Fig. 4, Fig. S5). Dissimilarities were captured primarily along PCoA Axis 1, where the contrast between waterborne communities and those on incubated particles was most apparent. While HDPE, TW, and Wood communities differed consistently from each other in their structure across all sites, dissimilarities between HDPE and TW communities appeared to diminish gradually along the gradient (Fig. S6). Concurrently, communities detected on these synthetic substrata grew more dissimilar to those on Wood, particularly upon transfer into coastal seawaters.

**Table 1:** Two-way PERMANOVA results showing the effects of Sampling Site and Sample Type on differences in community structure based on taxonomic and phylogenetic dissimilarity metrics.

Resemblance	Sources of variation	d.f.	SS	*pseudo F*	*p*_adj._	Sq. root
Bray Curtis	Sampling Site	3	49681	6.165	**0.0001**	28.689
	Sample Type	4	112540	9.170	**0.0001**	40.722
	Sampling Site × Sample Type	12	99353	3.642	**0.0001**	41.910
	Residuals	64	72201			33.588
	Total	83	352300			
Jaccard	Sampling Site	3	41148	5.738	**0.0001**	24.756
	Sample Type	4	81602	8.551	**0.0001**	33.407
	Sampling Site × Sample Type	12	97228	3.424	**0.0001**	37.995
	Residuals	64	142380			47.167
	Total	83	374690			
Weighted UniFrac	Sampling Site	3	1.054	29.434	**0.0001**	0.135
	Sample Type	4	2.400	50.254	**0.0001**	0.190
	Sampling Site × Sample Type	12	1.192	8.321	**0.0001**	0.147
	Residuals	64	0.764			0.109
	Total	83	5.723			
Unweighted UniFrac	Sampling Site	3	3.875	10.394	**0.0001**	0.250
	Sample Type	4	7.561	15.212	**0.0001**	0.329
	Sampling Site × Sample Type	12	6.544	4.389	**0.0001**	0.322
	Residuals	64	7.953			0.353
	Total	83	27.001			

*p* values were adjusted using Benjamini–Hochberg correction (*p*_adj._) and significance (*p* < 0.05) highlighted in bold.

*d.f.* degrees of freedom, *SS* sum of squares, *Sq. root* square root.

### Structural variations and turnover along a salinity gradient

**Fig. 5:**
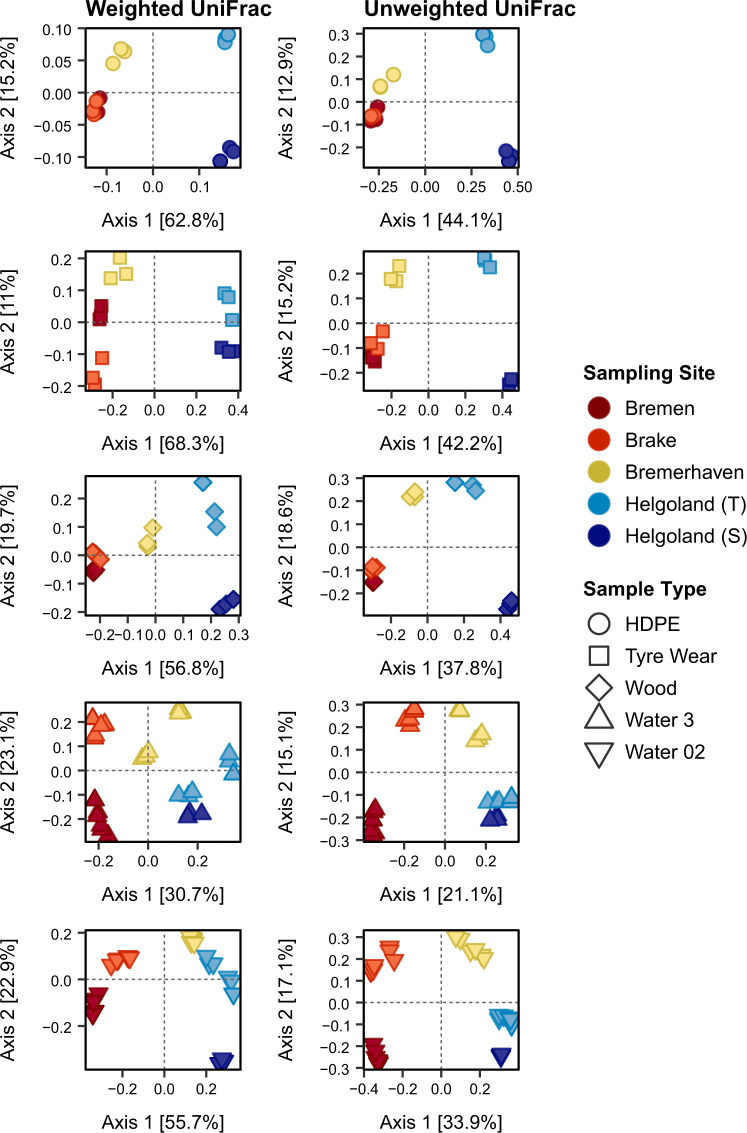
Phylogenetic structure of each community differed upon transfer along the salinity gradient. Phylogenetic dissimilarities were measured by weighted and unweighted UniFrac distances. Explained variation [%] along both Axes 1 and 2 are displayed in square brackets. Helgoland (T) and (S) represent samples from the transferred and stationary cages, respectively. For particle-associated (Water 3) and free-living (Water 02) waterborne communities, Helgoland (T) and (S) refer to surface waters sampled from the final offshore site before and after the incubation of the transferred and stationary cages, respectively, which were pooled together.

Communities on incubated particles adhered to different patterns of variation along the salinity gradient. The overall structure of HDPE and TW communities did not change significantly across riverine sites, except in abundances between certain sites (Table S9). Significant changes in their overall structure, however, were observed upon their transfer from brackish to coastal seawaters. The structure of Wood communities, analogous to particle-associated and free-living waterborne communities, differed significantly between most sites. As assumptions of multivariate homogeneity were violated, permuted values were validated through unconstrained ordinations, which reflected similar trends of variation. These were captured primarily along PCoA Axis 1 (Fig. 5, Fig. S7), where structural differences within each community by effect of the salinity gradient were evident, particularly along the transition zone between brackish and coastal seawaters.

**Fig. 6:**
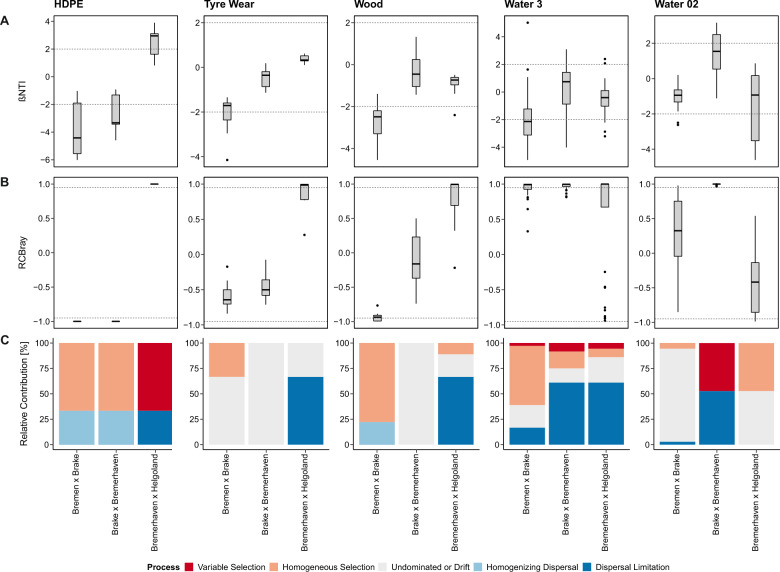
Assembly of communities was governed by different ecological processes for the different sample types. **A** The distribution of ßNTI values computed for each sample type across each pair of sites are presented in box plots along with their corresponding (**B**) RC_Bray_ values. **C** The relative contributions (%) of selection, dispersal or drift events that shaped each community across sites are shown in stacked bar plots. Helgoland (T) and (S) represent samples from the transferred and stationary cages, respectively. For particle-associated (Water 3) and free-living (Water 02) waterborne communities, Helgoland (T) and (S) refer to surface waters sampled from the final offshore site before and after the incubation of the transferred and stationary cages, respectively, which were pooled together.

Between riverine sites, the compositional turnover of HDPE communities was lower than expected by chance due to the selective pressures imposed by homogenous environmental conditions (homogenous selection, Fig. 6). TW communities, conversely, were undominated by selection or dispersal and experienced a non-significant turnover shaped by stochastic events across riverine sites. Wood communities, analogous to particle-associated and free-living waterborne communities, were shaped by a combination of deterministic and stochastic processes. Along the freshwater zone, the turnover of Wood communities was lower than expected by chance due to homogenous selection. Upon crossing the oligohaline zone into brackish waters, their turnover did not violate the null expectation, denoting equal pressures of selection and dispersal. Displacement from brackish to coastal seawaters resulted in an almost complete turnover of both HDPE and TW communities, where communities differed by a respective 75% and 72%. (Fig. S9 and Table S11**)**. Wood communities differed by an average of 51% and were slightly more nested, with a respective 10% and 15% of the taxonomic and phylogenetic dissimilarities observed along this stretch of the gradient attributed to species loss/gain. For HDPE communities, displacement across these transitional waters resulted in a high compositional turnover due to a shift in selective pressures (variable selection). The phylogenetic turnover of TW and Wood communities, conversely, did not deviate from the null expectation, rather a significant turnover was observed in their taxonomy as a product of low dispersal rates (dispersal limitation).

*Alphaproteobacteria* contributed the most to differences within HDPE communities along the gradient (Table S11), namely from a general increase in *Rhodobacteraceae*. The recruitment of *Thiotrichaceae* and *Flavobacteriaceae* upon transfer into coastal seawaters additionally contributed to the large compositional differences observed along this stretch of the gradient. Variations within Wood communities were similarly driven by an increase in *Rhodobacteraceae* and *Sphingomonadaceae* across riverine sites but with a near two-fold decline in their counts upon transfer into coastal seawaters. A large increase in *Flavobacteriaceae* was also observed upon the transfer of Wood from freshwater to brackish and coastal seawaters. Compositional differences within TW communities along the gradient were attributed chiefly to *Gammaproteobacteria*, represented mostly by changes in the SS1-B-06-26 family. Members of SS1-B-06-26 increased gradually across riverine sites but declined upon transfer into coastal seawaters, where the recruitment of *Flavobacteriaceae* and *Pseudohongiellaceae* was observed instead.

The structure of transferred communities upon reaching the final offshore site differed significantly from that of their stationary equivalents which remained offshore throughout the experiment (Tables S12 and S13). These dissimilarities were attributed almost completely to species replacement, suggesting stark compositional distinctions between corresponding communities (Fig. S9). Transferred HDPE and TW communities differed in their composition from their stationary counterparts by an average of 60% and 65%, respectively (Table S11). For HDPE, these differences stemmed mostly from a greater abundance of *Rhodobacteraceae* and *Thiotrichaceae* detected on transferred particles. Transferred and stationary TW particles were distinguished predominantly by lower counts of SS1-B-06-26 detected on the former which instead harboured a greater abundance of *Pseudohongiellaceae* and *Flavobacteriaceae*. Only 14% and 15% of the total ASVs detected on transferred HDPE and TW at the final site, respectively, were displaced from riverine sites and absent from their stationary equivalents. ASVs transferred on HDPE consisted mostly of *Anaerolineae* and *Deinococci* variants, along with classes that were entirely absent from their stationary counterparts such as *Nitrospira* and *Acidobacteria* Subgroup 6. Variants of the SS1-B-06-26 family made up the majority of the transferred ASVs on TW, along with *Alteromonadales* and *Pseudomonadales*, which were undetected on their stationary equivalents.

Relative to the synthetic substrata, differences between transferred and stationary Wood particles were the greatest. At the final site, both transferred and stationary Wood communities differed in their composition by an average of 93%, due predominantly to greater proportions of *Rhodobacteraceae* and *Sphingomonadaceae* detected on stationary Wood particles. Transferred Wood particles instead harboured greater counts of *Flavobacteriaceae*. Wood, however, displaced the greatest proportion of ASVs along the gradient relative to synthetic substrata, with 26% of their total ASVs detected in the offshore site having originated from riverine sites. These consisted predominantly of variants of *Rhodobacteraceae*, *Sphingomonadaceae*, and *Flavobacteriaceae*.

## Discussion

The potential of MP to raft bacteria across different aquatic systems was investigated in this study through a sequential incubation experiment performed along a salinity gradient. Bacterial biofilms detected on synthetic and natural substrata were analysed and compared to each other as well as to surrounding waterborne communities. In our study, HDPE communities were significantly more diverse than other sample types except particle-associated waterborne communities, to which their overall diversity was largely comparable. These findings are consistent with those previously reported of plastic sampled from coastal seawaters [14, 15]. Other similar studies conversely reported polyethylene communities to be less diverse in comparison to those in surrounding waters [8, 46]. While the comparability of studies remains limited due to their varied designs, we posit these contrasting findings to stem from the variability of environments studied. The formation of biofilm communities, though largely governed by the physicochemical properties of the substrata, are additionally dictated by environmental cues such as hydrodynamic forces [6], which may differ drastically across environments as diverse as those of coastal waters.

Given the hydrophobicity of both HDPE and TW surfaces [47, 48], similar patterns of diversity among their respective communities were expected. Both communities, however, were diametrically opposed, with HDPE communities representing the highest diversity and TW communities the lowest. We postulate the low diversity of TW communities to be the product of the substratum’s complex composition. TW consist of a blend of natural and synthetic rubber polymers, reinforcers (e.g., carbon black, silica), softeners (petroleum process oils), as well as vulcanizing agents and accelerators (e.g., zinc oxide, sulfur compounds) [49, 50]. Within aqueous solution, certain constituents, namely the polycyclic aromatic hydrocarbons (PAH) present in the softeners and heavy metals used in the vulcanization process, have been reported to leach from the rubber matrix into surrounding environments [51, 52]. Exposure to a combination of PAHs and heavy metals was shown by Thavamani et al. [53] to alter bacterial populations and result in a lower observed diversity. In our study, *Gammaproteobacteria* consistently made up a dominant proportion of TW communities, comprising significantly greater counts of hydrocarbon-degrading families of the *Oceanospirillales* order, such as *Oleiphilaceae*, *Pseudohongiellaceae*, and SS1-B-06-26 than other sample types [54–56]. Significantly greater counts of other hydrocarbon-utilizing *Gammaproteobacteria*, such as *Porticoccaceae*, *Immundisolibacterales*, and *Methylophilaceae* [57–59] were also detected. Hydrocarbonoclastic *Oceanospirillales* have previously been reported to occur on MP of different polymer types [60–63]. To the best of the authors’ knowledge, however, there exists only one published report on the bacterial colonization of tyre particles. In their study, Wang et al. [48] investigated the biofilms of tyre particles sampled from urban water bodies and reported a similar dominance of *Gammaproteobacteria*. *Aquabacterium*, a genus of *Betaproteobacteria* reportedly capable of hydrocarbon utilization, was also detected. The pronounced concentration of hydrocarbonoclastic bacteria consistently detected along the salinity gradient in our study, with the collective capability to degrade linear, branched, or aromatic hydrocarbons, suggests that TW act as a carbon source for a selective consortium of core bacteria. While further investigations are required to substantiate this thesis, the detection of *Oleiphilus* and *Porticoccaceae* strongly support our hypothesis as these taxa have been categorized as obligate hydrocarbon degraders and rely on aliphatic hydrocarbons or mono- and polycyclic aromatics as their primary sources of energy [54, 57, 64].

Consistent with published studies, *Alphaproteobacteria* and *Gammaproteobacteria* were the two most dominant classes detected of all sample types [8, 15, 65]. *Alphaproteobacteria* were significantly more abundant on Wood and were composed predominantly of *Rhodobacteraceae* and *Sphingomonadaceae*. These families are among the most dominant and well-known surface colonizers reported of aquatic environments [6, 66] and as such also represented a large proportion of HDPE communities in this study. The composition of HDPE communities, however, were more heterogenous relative to TW and Wood and were distinguished by the more minor fractions of their overall community. The occurrence of many less abundant taxa leads one to speculate that the substratum does not strongly select for specific bacterial consortia. A similar conjecture was made in a meta-analysis by Oberbeckmann and Labrenz [19], where most polyethylene-specific taxa were represented by low-abundance operational taxonomic units and interpreted as a lack of polymer specificity. The specificity of MP as a substrate, however, remains heavily contended and should be better addressed by further functional analyses of their biofilms before more conclusive inferences can be drawn.

Along the salinity gradient, all sample types differed significantly from each other in their structure. The most pronounced differences were observed between communities on incubated particles and those within surrounding waters, which differed consistently from each other along the gradient. While dominated by similar bacterial classes, only a minor proportion of the total ASVs on incubated particles were also found in surrounding waters. These findings, though consistent with published studies [9, 12], contradict the expectation that waterborne communities serve as a source for the biofilms that establish on substrata surfaces in aquatic environments. These striking structural dissimilarities, however, were explained by Jousset et al. [67] to stem from the fact that biofilm communities recruit from the surrounding rare biosphere rather than from its most dominant members. As rare taxa often comprise a minor fraction of the overall community, their detection within waterborne communities can be difficult due to the limitations of existing sequencing technologies, resulting in a seemingly minimal overlap between biofilm and waterborne communities [68].

Distinct patterns of variation were also observed within each community along the salinity gradient. For HDPE, these changes were driven predominantly by environmental selection. While seemingly susceptible to environmental filtering on a local scale, TW and Wood communities were governed more by stochastic events on a regional scale. The opposing local and regional patterns observed among TW and Wood communities likely indicate that, while community assembly is shaped by deterministic factors, their turnover is more strongly influenced by stochastic drift or dispersal events [69]. Upon transfer into coastal seawaters, communities on all three substrata experienced significant changes in their structure, with an almost complete turnover observed among HDPE and TW communities. This did not defy expectation as salinity has been identified as one of the major factors that shape microbial communities [70]. In our study, the salinity shift observed along the transition from brackish to coastal seawaters imposed a strong selective effect on HDPE communities, resulting in a greater-than-expected turnover, but did not overwhelm the influence of stochastic processes on TW and Wood communities. Instead, a significant turnover was observed in their taxonomy due to a limited exchange with surrounding source communities – a process referred to as dispersal limitation. Neutral theory, the framework under which dispersal limitation falls, assumes demographic stochasticity among communities. Member taxa, convergent in their functional traits, share identical levels of fitness and their compositional dynamics are consequently the product of random events [71]. Such phenotypic similarities mirror the less heterogeneous nature of TW and Wood communities, where *Gammaproteobacteria* and *Alphaproteobacteria*, comprised more than 50% of their respective overall populations.

A gradual convergence in the overall structure of HDPE and TW communities was observed along the salinity gradient. This might first be explained by the coalescing effect that confluent water bodies of differing salinities reportedly have on microbial communities. In a study by Rocca et al. [72], communities translocated from freshwater and marine environments into brackish waters converged in their structure towards that of marine microbiomes, with a substantial loss of freshwater taxa. This was demonstrated, in our study, by a significant turnover of HDPE, TW, and Wood communities upon transfer across transitional waters in addition to the recruitment and increasing dominance of new member taxa, such as *Flavobacteriaceae* and *Thiotrichaceae*, which have been reported within estuarine environments and described as marine bacterioplankton with an adaptive tolerance to lower salinities [73]. A compounding factor to salinity in driving such structural convergence is the age of the biofilms. A similar confluence over time was reported by Pinto et al. [62], who observed a diminishing distinction between different plastic- and glass-associated communities proportional to incubation time. As biofilms mature, the densities of communities grow and the influence of the substrate itself recedes in its importance to the differentiation of recruited taxa due to limited access to the substratum surface. Consequently, the organic layer established on these substrata are marked by similar functional niches filled by increasingly resemblant bacteria [68]. Wood communities, however, grew more distinct in their structure from those on synthetic particles upon transfer into coastal seawaters and experienced a turnover more resultant of species loss/gain. The properties of wooden substrates are nonetheless quite discrete from those of synthetic substrata. While the density of mature biofilms on surfaces as inert as HDPE and TW limits substrate utilization by late colonizers, the relatively greater potential of Wood for degradation has been posited to provide degradation products to external layers of the biofilm by lateral diffusion [68, 74]. Consequently, structural variations within Wood communities were consistently dictated by a few dominant taxa throughout the salinity gradient.

This was also evident when considering the transport potential of the different substrata. Wood successfully displaced a greater proportion of their total ASVs along the salinity gradient than HDPE or TW. ASVs transported on Wood, however, consisted of variants of the most dominant taxa detected of Wood communities whereas the synthetic substrata additionally rafted taxa completely undetected on their stationary counterparts on the class and order level. The recruitment and successful displacement of *Nitrospira* and *Acidobacteria* Subgroup 6 on HDPE from freshwater to coastal seawaters is not surprising given their reportedly ubiquitous nature across aquatic environments [75, 76]. More striking is the transportation of *Pseudomonadales* and *Alteromonadales* on TW, which were respectively recruited from freshwater and brackish waters. These orders consisted mostly of an unclassified genus of *Moraxellaceae* and *Aestuariibacter*, both of which have been detected within riverine and coastal environments [63, 77]. As *Aestuariibacter* are known hydrocarbon-degrading bacteria [63], their detection on TW further solidifies our thesis on the selectivity of TW as a substrate. Members of the *Moraxellaceae* family, however, bear particular clinical importance. In addition to several species having been identified as causal agents for a range of human and animal infections, *Moraxellaceae* are often reported to harbour antibiotic resistance genes [78–80]. These findings offer interesting opportunities for further research into the functionality of these bacteria and their dynamics along a salinity gradient to more completely assess the threat that MP might pose in successfully displacing potentially harmful species across different aquatic environments.

## Conclusion

This study offers a first look into the dynamics of bacterial communities on the surfaces of synthetic and natural substrata along a salinity gradient. In addition to providing a deeper understanding of MP biofilms, we show that the displacement of these substrata from riverine to coastal seawaters elicits a high turnover of communities, suggesting an unlikely translocation of species across these aquatic environments on a substantial scale. Nonetheless, HDPE and TW were found capable of rafting certain freshwater taxa into seawater environments, some of which may be harmful. Putatively, we also demonstrate the specificity of TW as a substrate for bacteria and present exciting new avenues for further research into the functionality of these biofilms to more fully assess the role of MP as transport vectors for bacteria and their potential threats.

## Supplementary information

Supplementary Material Guide

Supplementary Material

Supplementary Table 1

Supplementary Table 2

Supplementary Table 3

Supplementary Table 8

Supplementary Table 9

Supplementary Table 10

Supplementary Table 11

Supplementary Table 13
