# Supplementary material for: The travelling particles: community dynamics of biofilms on microplastics transferred along a salinity gradient
Source: ISME Commun. 2022 Apr 11;2:35. doi: 10.1038/s43705-022-00117-4 (PMC9723596; doi:10.1038/s43705-022-00117-4)
Supplement: Supplementary file 1 — Supplementary Material Guide [file 43705_2022_117_MOESM1_ESM.docx]

**Supplementary Guide for**: The Travelling Particles: Community Dynamics of Biofilms on Microplastics Transferred along a Salinity Gradient

Jessica Song^1^, Lukas Beule^2^, Elanor Jongmans-Hochschulz^1^, Antje Wichels^1^, Gunnar Gerdts^1^

^1^Department of Microbial Ecology, Biologische Anstalt Helgoland, Alfred Wegener Institute Helmholtz Centre for Polar and Marine Research, 27498 Helgoland, Germany

^2^Julius Kühn Institute—Federal Research Centre for Cultivated Plants, Institute for Ecological Chemistry, Plant Analysis and Stored Product Protection, Königin-Luise-Strasse 19, 14195 Berlin, Germany

****Correspondence***: Jessica Song, jessica.song@awi.de

1. **Supplementary Information**: A merged PDF of Supplementary Methods, Supplementary Figures (images attached separately as single PDFs), and Supplementary Tables.
2. **Fig. S1**: A PDF document showing a richness curve (caption in **Supplementary Information**)
3. **Fig. S2**: A PDF document showing a differential heat tree comparing taxa detected on different sample types (caption in **Supplementary Information**)
4. **Fig. S3**: A PDF document showing a stacked bar plot of the relative abundances of detected bacterial orders per sample type at each site (caption in **Supplementary Information**)
5. **Fig. S4**: A PDF document showing the number of shared and unique ASVs for each sample type in the form of an UpSet plot (caption in **Supplementary Information**)
6. **Fig**. **S5**: A PDF document showing the PCoA plots generated based on Bray-Curtis and Jaccard dissimilarity between the different sample types at each site (caption in **Supplementary Information**)
7. **Fig. S6**: A PDF document showing box plots displaying the patterns of dissimilarity between each pair of substrata across the different sites (caption in **Supplementary Information**)
8. **Fig. S7**: A PDF document showing the PCoA plots generated based on Bray-Curtis and Jaccard dissimilarity within each sample type across the different sites (caption in **Supplementary Information**)
9. **Fig. S8**: A PDF document showing Mantel correlograms generated through phylogenetic signal calculations (caption in **Supplementary Information**)
10. **Fig. S9**: A PDF document showing stacked bar plots of the taxonomic and phylogenetic dissimilarities within each substratum across the different sites partitioned into components of turnover and nestedness.
11. **Table S1**: An Excel file (comprised of two sheets) showing background information on incubation sites, samples collected, and DNA extraction.
12. **Table S2**: An Excel file showing the mean relative abundances of overall detected bacterial phyla for each sample type.
13. **Table S3**: An Excel file showing the mean relative abundances of overall detected bacterial classes for each sample type.
14. **Table S8**: An Excel file showing the results of a post-hoc two-way PERMANOVA and PERMDISP of different sample types at each site based on taxonomic and phylogenetic dissimilarity metrics.
15. **Table S9**: An Excel file showing the results of a post-hoc two‑way PERMANOVA and PERMDISP of each sample type across sites based on taxonomic and phylogenetic dissimilarity metrics.
16. **Table S10**: An Excel file (comprised of five sheets) showing the ßNTI and RC_Bray_ matrices computed for each sample type across the different sites.
17. **Table S11**: An Excel file (comprised of five sheets) showing results of SIMPER analyses conducted for each sample type across sites.
18. **Table S13:** An Excel file showing the results of a post-hoc one-way PERMANOVA and PERMDISP of samples collected at the final offshore site from the transferred and stationary cages and surrounding waters based on taxonomic and phylogenetic dissimilarity metrics.
