# Supplementary material for: The travelling particles: community dynamics of biofilms on microplastics transferred along a salinity gradient
Source: ISME Commun. 2022 Apr 11;2:35. doi: 10.1038/s43705-022-00117-4 (PMC9723596; doi:10.1038/s43705-022-00117-4)
Supplement: Supplementary file 2 — Supplementary Material [file 43705_2022_117_MOESM2_ESM.pdf]

Supplementary Information for: The Travelling Particles: Community Dynamics of Biofilms on Microplastics Transferred along a Salinity Gradient

Jessica Song[1], Lukas Beule[2], Elanor Jongmans-Hochschulz[1], Antje Wichels[1], Gunnar Gerdts[1]

[1]Department of Microbial Ecology, Biologische Anstalt Helgoland, Alfred Wegener Institute Helmholtz Centre for Polar and Marine Research, 27498 Helgoland, Germany

[2]Julius Kühn Institute—Federal Research Centre for Cultivated Plants, Institute for Ecological Chemistry, Plant Analysis and Stored Product Protection, Königin-Luise-Strasse 19, 14195 Berlin, Germany

****Correspondence***: Jessica Song, jessica.song@awi.de

Supplementary Methods

Detailed description of sample collection

Following each incubation, particles and surrounding waters were sampled in triplicate. Particles were stored in 1 L Kautex bottles filled with water from each respective site while water samples were stored in 1L Nalgene bottles. All sampling events were conducted at outgoing tide and samples collected were transported back to the laboratory in a cooling box (approx. 4 – 5 °C) on the same day for immediate processing. After each sampling event, the particles were transported to the next site under *in situ* conditions, where the cage containing the particles was placed into a transport container filled with water from the respective incubation sites and kept at a temperature of approximately 4 – 5 °C with ice packs.

Detailed description of sample preparation and amplicon sequencing

All particles within each capsule were transferred into 40 ml of 1 × phosphate buffered saline (PBS) and vortexed three times to remove any loosely-attached material, following which total biofilm DNA was extracted. The concentration of extracted DNA was quantified using the Quant-iT™ PicoGreen™ dsDNA Assay Kit (Invitrogen, Waltham, MA, USA) measured with a Tecan Infinite M200 PRO NanoQuant microplate reader (Tecan Trading AG, Switzerland).

PCR was performed in 20 µL reaction volumes containing 1 × MyTaq buffer (Bioline GmbH, Luckenwalde, Germany), 1.5 u MyTaq DNA polymerase (Bioline GmbH, Luckenwalde, Germany), 15 pmol of each forward and reverse primer (with a 10-nt barcode sequence at the 5’-end of each primer), 2 µl of BioStabII PCR Enhancer (Sigma-Aldrich Co.), and 1 µL template DNA. Thermocycling conditions were as follows: 60 s initial denaturation at 96 °C, followed by 30 cycles of denaturation for 15 s at 96 °C, annealing for 30 s at 55 °C, and elongation for 90 s at 70 °C. Amplicons were checked via gel electrophoresis, pooled, and purified using Agencourt AMPure XP beads (Beckman Coulter, Inc., IN, USA). An additional purification step was performed using MiniElute columns (QIAGEN GmbH, Hilden, Germany). Illumina libraries were constructed from the purified amplicon pools using the Ovation Rapid DR Multiplex System 1-96 (NuGEN Technologies, Inc., CA, USA) and subject to 300 bp paired-end read sequencing using an Illumina MiSeq (V3 chemistry).

Detailed description of 16S amplicon sequence processing

Forward and reverse reads were truncated at 205 bp and 245 bp, respectively, using the DADA2 plugin on the QIIME2 platform to remove low quality bases (median score < 30). Reads were dereplicated at 100 % sequence identity and denoised using a parametric error model. The resulting reads were then merged and a global alignment performed for *de novo* chimera detection and removal.

Details on differential abundance testing

Statistical tests were performed using the *calc_diff_abund_deseq2* function offered by the *metacoder* package, which employs a negative binomial model and Wald test for hypothesis testing to identify differentially abundant taxa between sample types. The test was performed on raw read counts and hypothesis testing conducted at a significance level of $\alpha = 0.05$, with a Benjamini-Hochberg correction applied to obtained *p*-values.

Details on permutational analysis of variance (PERMANOVA)

Variation partitioning was performed at a significance level of $\alpha = 0.05$ using type III sum of squares and *p*-values obtained using 9999 permutations except for samples where too few possible permutations were available, during which Monte Carlo simulations were employed. Permuted *p*-values were adjusted using Benjamini-Hochberg correction.

Details on quantitative process estimates using null models

The compositional turnover of the different communities between sites was quantified through pairwise comparisons based on the ß-nearest taxon index (ßNTI). Values obtained were then compared against a null distribution, representative of stochastically-assembled communities, and differences measured in standard deviation units. Positive and negative deviations from the null distribution (ßNTI > 2, ßNTI < -2) are indicative of variable and homogenous selection, respectively. The former is defined as a high compositional turnover elicited by a shift in selective pressures whereas the latter suggests a low turnover due to exposure to relatively unvarying environmental conditions. Environmental selection is ruled out as the dominant assembly process when no significant deviations from the randomly-assembled communities are observed (-2 < ßNTI < 2), for which dispersal or ecological drift is inferred to be the primary influence. To estimate the relative contributions of these stochastic processes, the Raup-Crick metric [43] was calculated based on Bray-Curtis dissimilarities (RC_{bray}) as a non-phylogenetic analogue to the ßNTI for the comparison of observed and expected taxonomic turnover. Similarly, deviations from the null expectation were suggestive of dispersal limitation ($RC_{bray} > 0.95$) or homogenizing dispersal ($RC_{bray} > -0.95$). Dispersal limitation describes a scenario where communities drift apart due to a low dispersal rate, resulting in a high compositional turnover, while homogenizing dispersal increases compositional similarities as a consequence of high dispersal rates overwhelming the effects of environmental selection. The final scenario, represented by values that fall within the null distribution ($-0.95 < RC_{bray} < 0.95$), indicates a turnover undominated by selection or dispersal, otherwise referred to as ecological drift.

Supplementary Figures

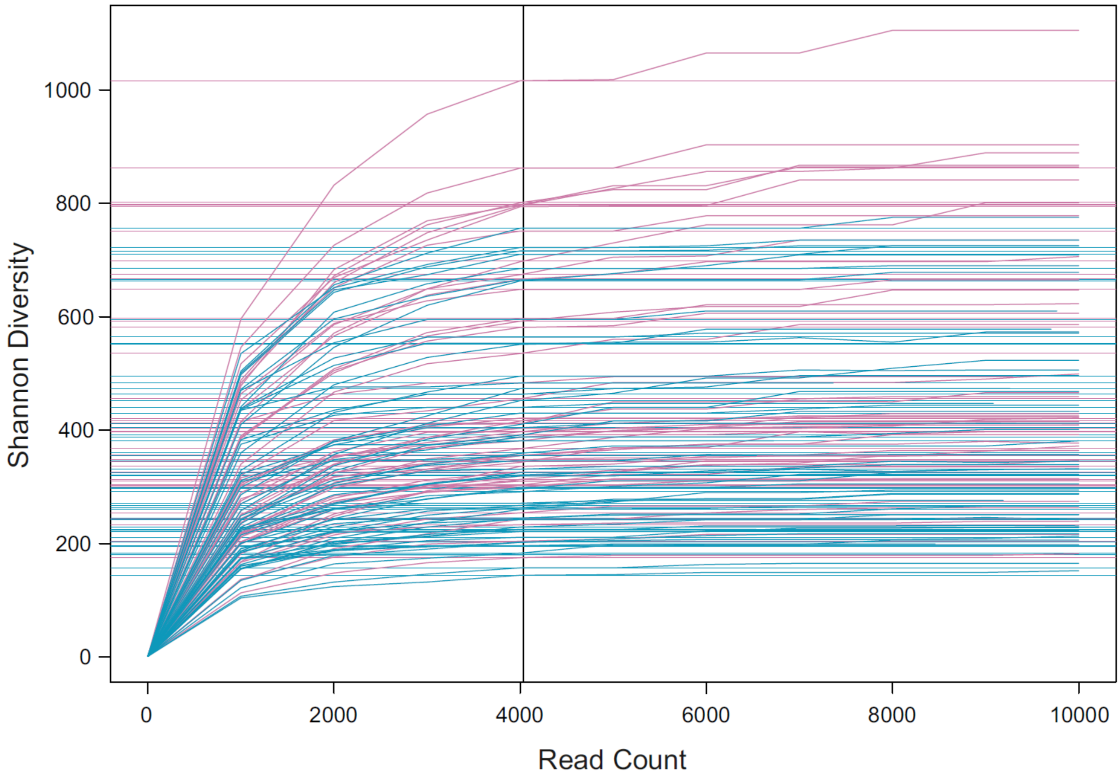

Fig. S1 Species richness curve generated from normalized reads based on Shannon's diversity index displaying a sufficient sampling depth. Reads were normalized to the lowest read count detected, resulting in 4036 reads per sample (indicated by the black line).

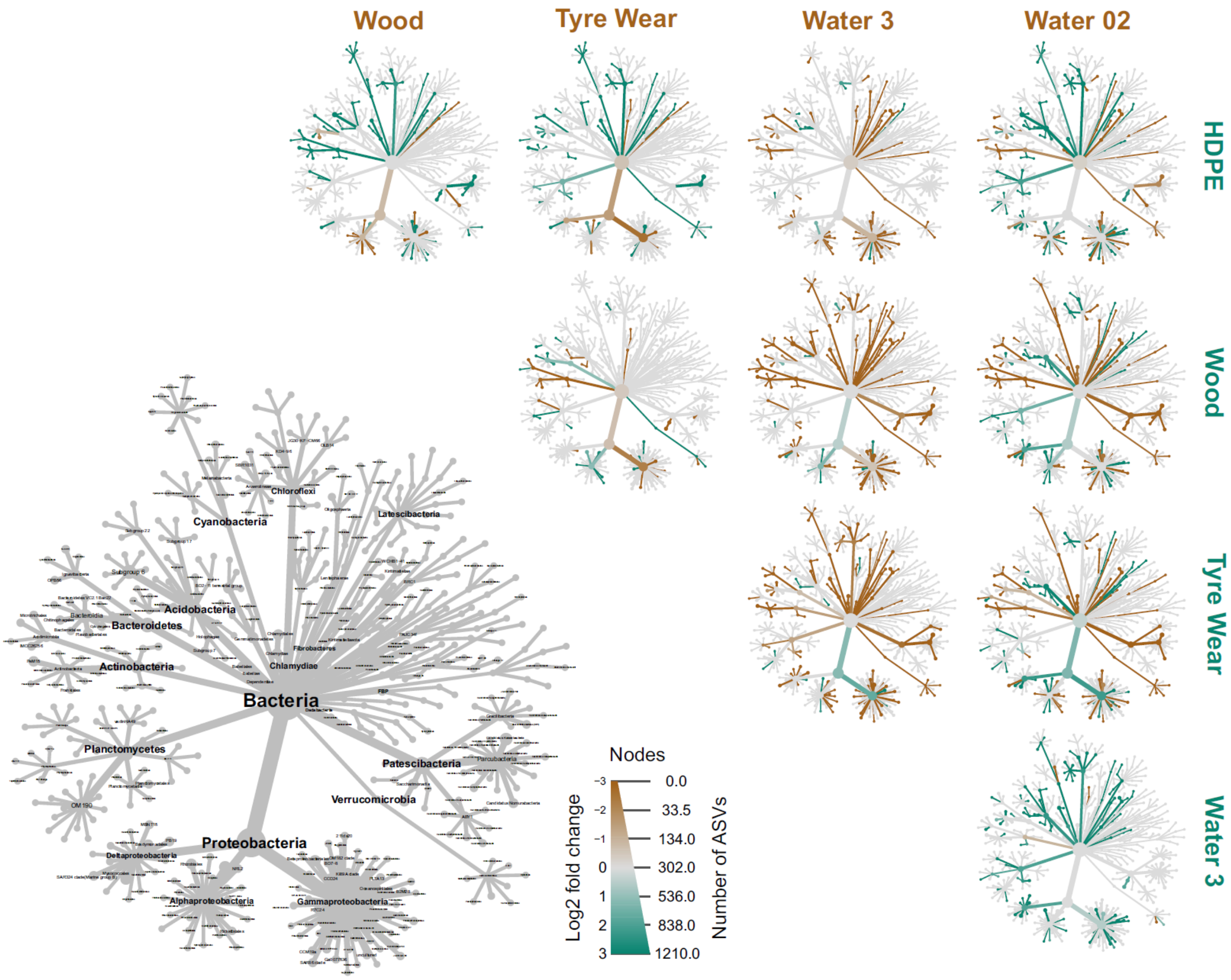

Fig. S2 Heat tree showing significant differences in the median read counts of taxa within total communities detected of different sample types. The trees are collapsed at the order level and taxa labels provided in the grey tree on the lower left. Green highlighted branches are indicative of a greater abundance of a taxon in the sample type listed in the row while taxa that are more abundant in the sample type listed in the column are highlighted in brown. Node sizes are representative of the number of ASVs detected per taxon. Water 3: particle-associated waterborne communities; Water 02: free-living waterborne communities.

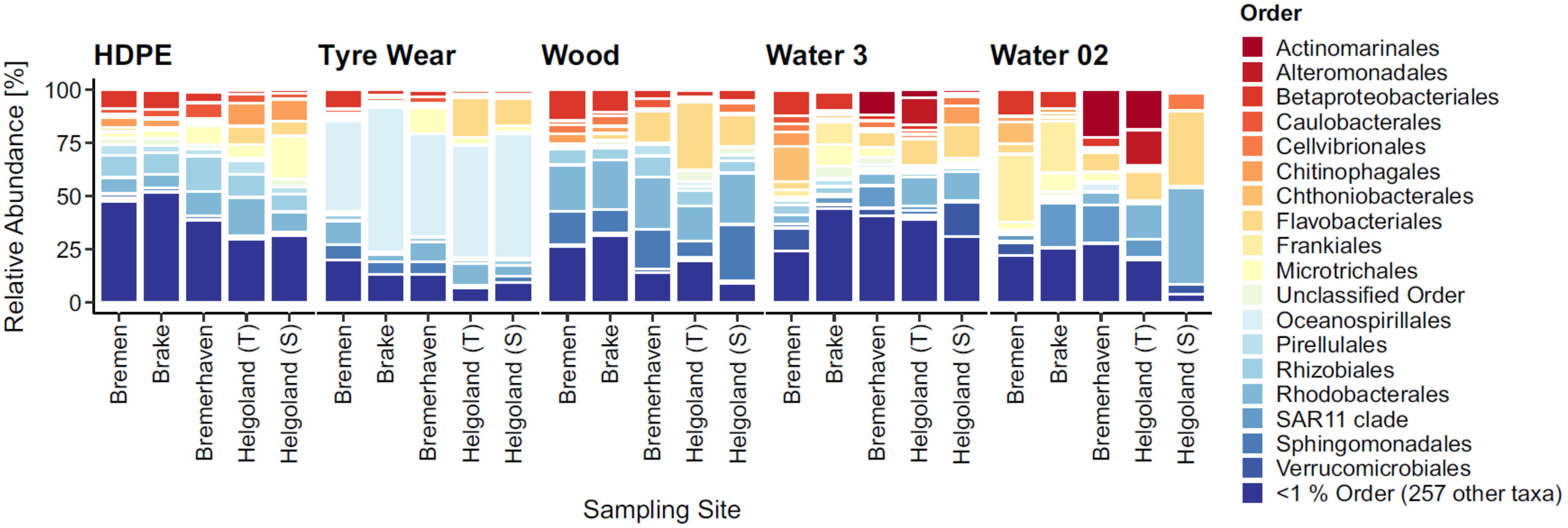

Fig. S3 Relative proportions of bacterial orders detected for each sample type at each site. All orders with a mean relative abundance below 1% were grouped into one category (<1% Order). Helgoland (T) and (S) represent samples from the transferred and stationary cages, respectively. For particle-associated (Water 3) and free-living (Water 02) waterborne communities, Helgoland (T) and (S) refer to surface waters sampled from the final offshore site before and after the incubation of the transferred and stationary cages, respectively, which were pooled together.

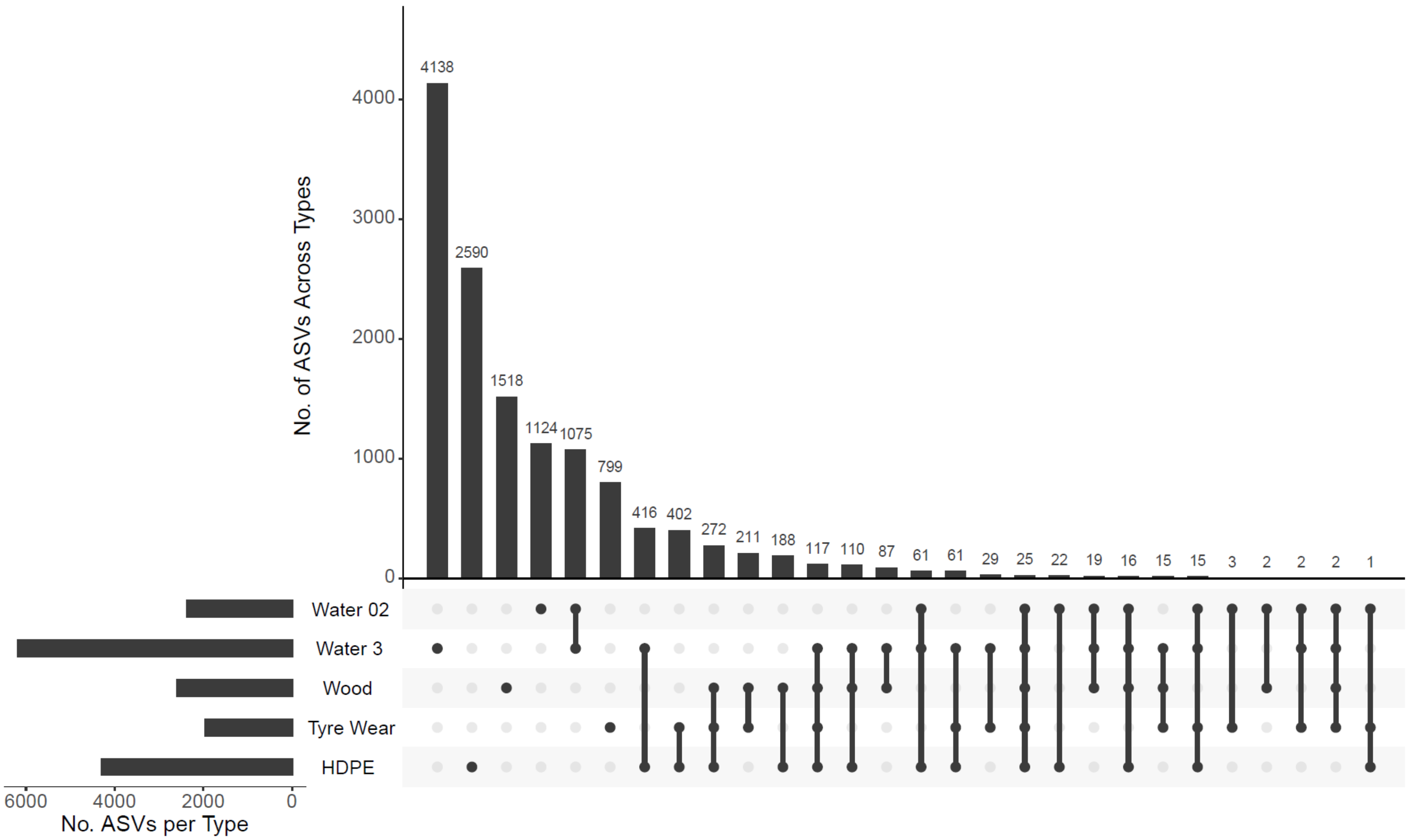

Fig. S4 UpsetR plot displaying the total number of detected ASVs shared and unique to each sample type. The total number of ASVs detected for each sample type are shown in the horizontal bars to the left while vertical bars represent the number of ASVs shared or unique to each type highlighted by black nodes. Water 3: particle-associated waterborne communities; Water 02: free-living waterborne communities.

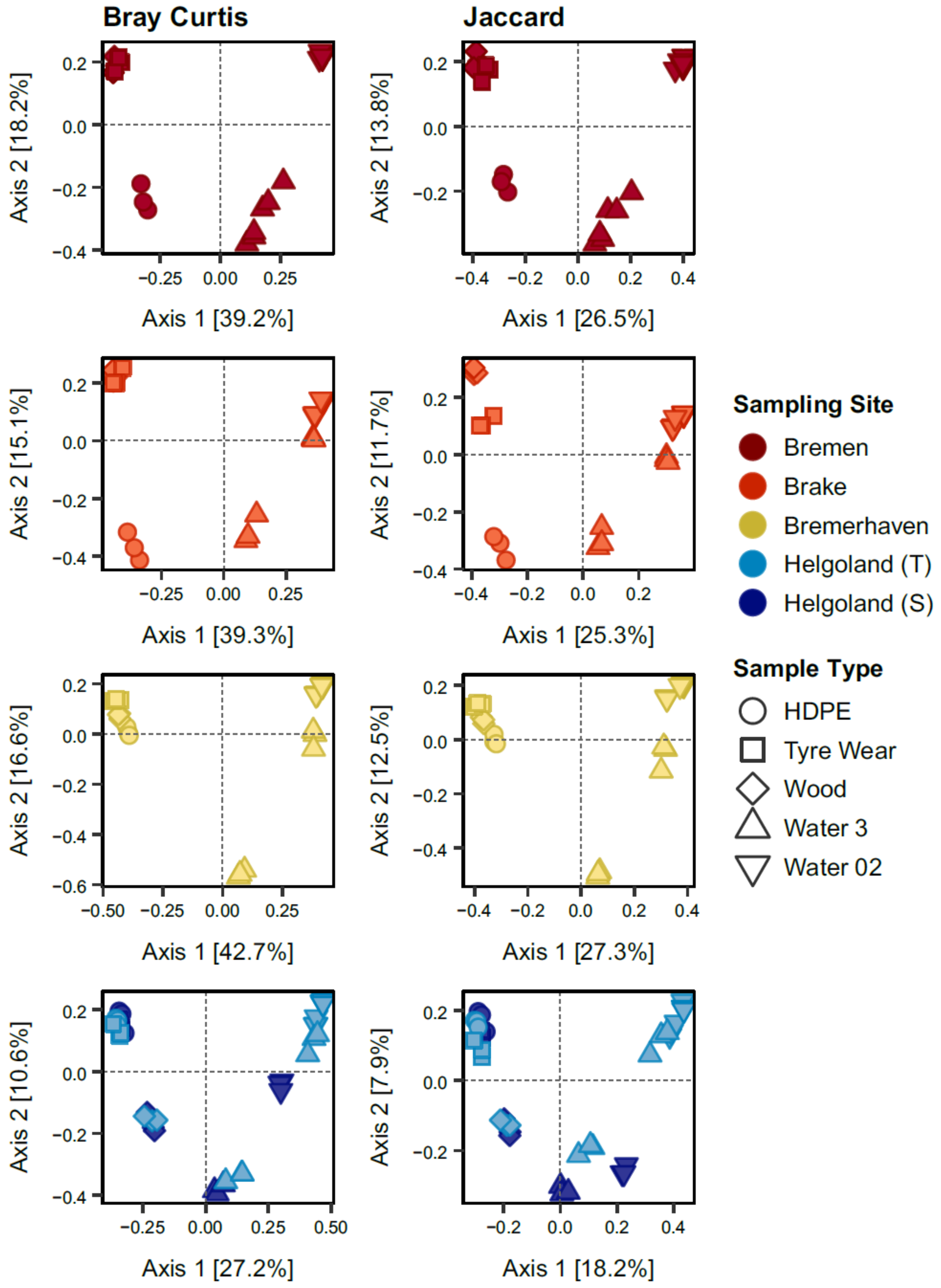

Fig. S5 Principal Coordinate Analysis (PCoA) plots showing the taxonomic dissimilarities between the different sample types at each site based on Bray-Curtis and Jaccard indices. Explained variation [%] along both Axes 1 and 2 are displayed in square brackets. Helgoland (T) and (S) represent samples from the transferred and stationary cages, respectively. For particle-associated (Water 3) and free-living (Water 02) waterborne communities, Helgoland (T) and (S) refer to surface waters sampled from the final offshore site before and after the incubation of the transferred and stationary cages, respectively, which were pooled together.

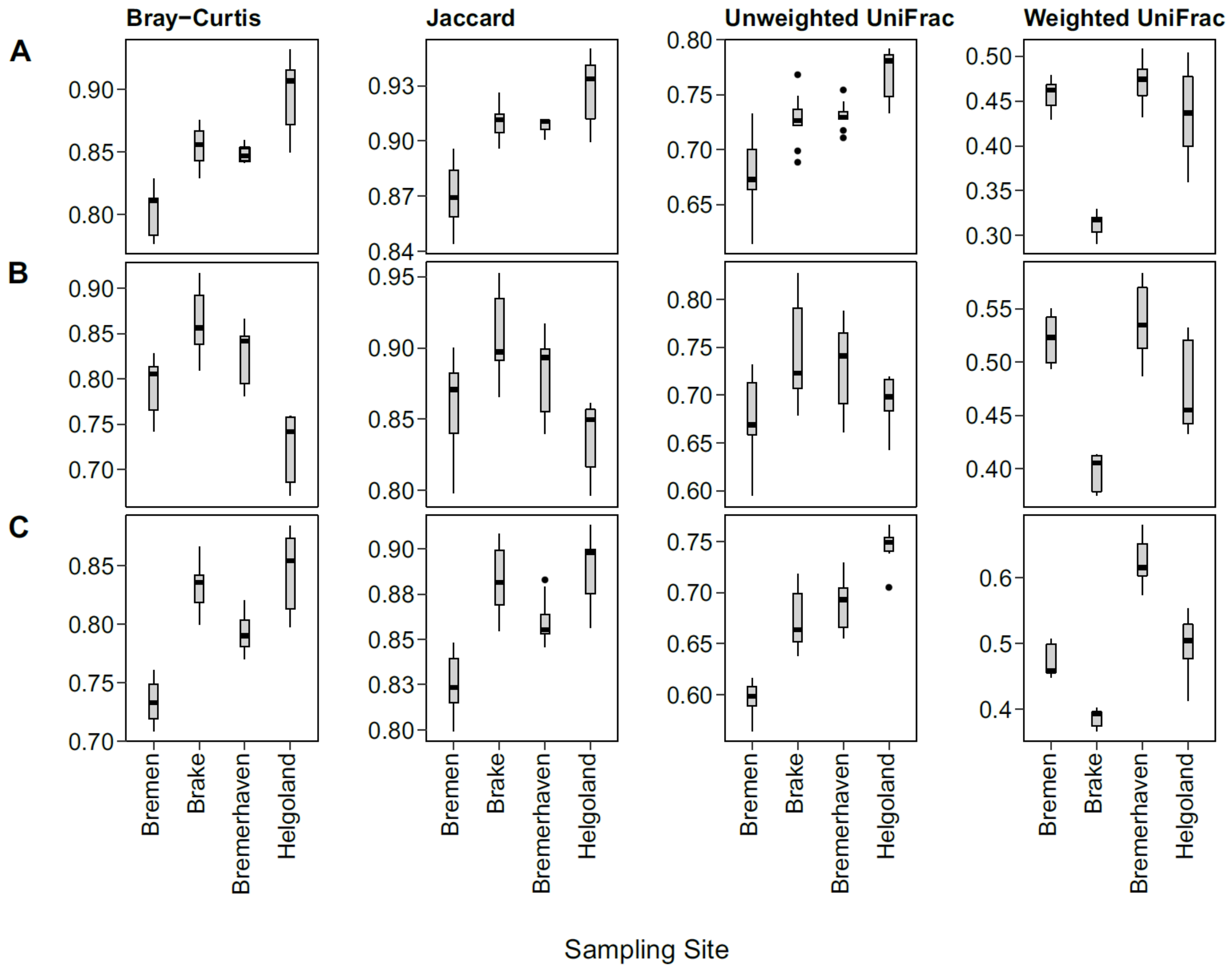

Fig. S6 Box plots showing the dissimilarity between A) HDPE and Wood, B) HDPE and Tyre Wear, and C) Wood and Tyre Wear at each site based on taxonomic (Bray-Curtis and Jaccard) and phylogenetic (weighted and unweighted UniFrac) dissimilarity metrics.

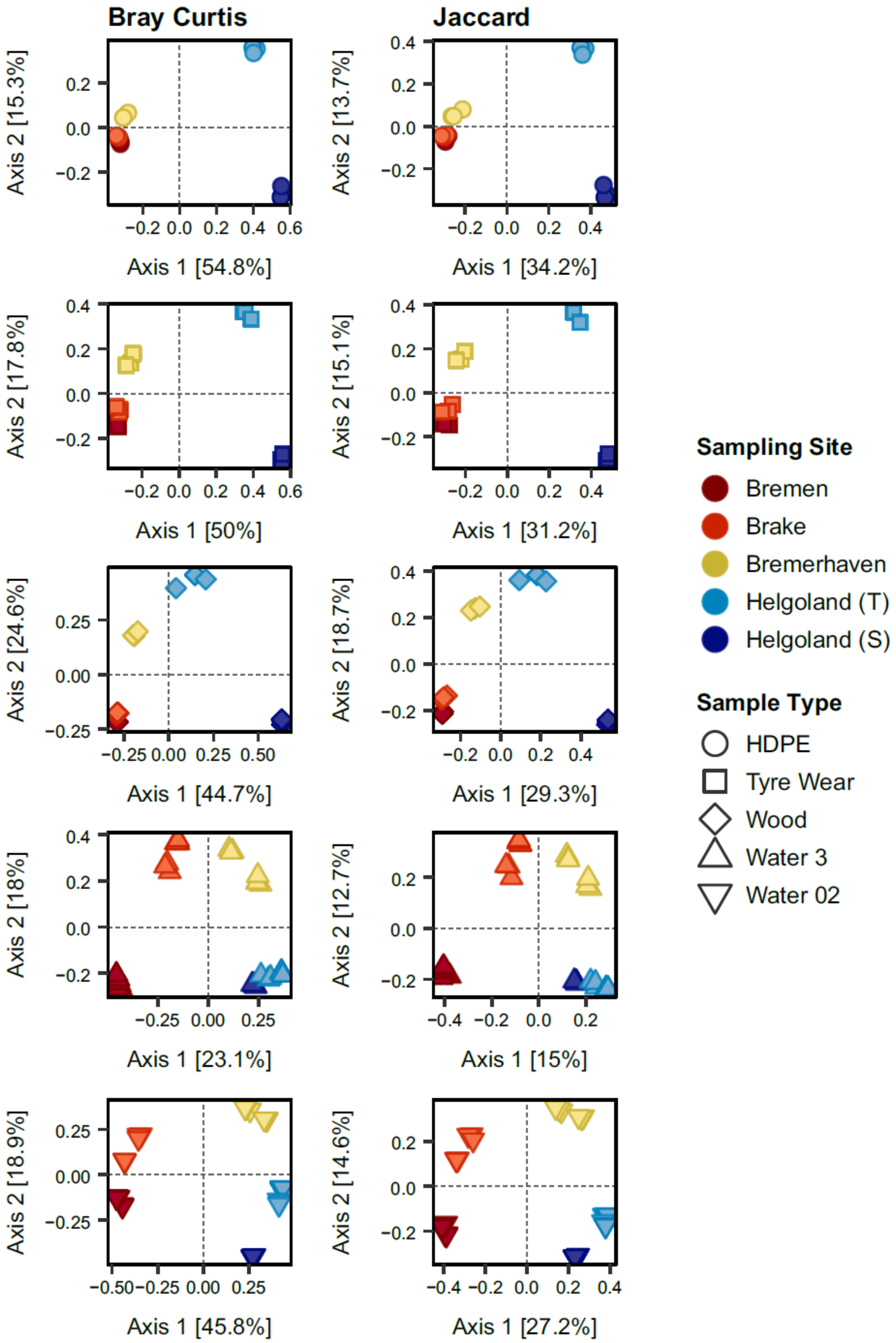

Fig. S7 Principal Coordinate Analysis (PCoA) plots showing the taxonomic dissimilarities observed within each sample type across the different sites based on Bray-Curtis and Jaccard indices. Explained variation [%] along both Axes 1 and 2 are displayed in square brackets. Helgoland (T) and (S) represent samples from the transferred and stationary cages, respectively. For particle-associated (Water 3) and free-living (Water 02) waterborne communities, Helgoland (T) and (S) refer to surface waters sampled from the final offshore site before and after the incubation of the transferred and stationary cages, respectively, which were pooled together.

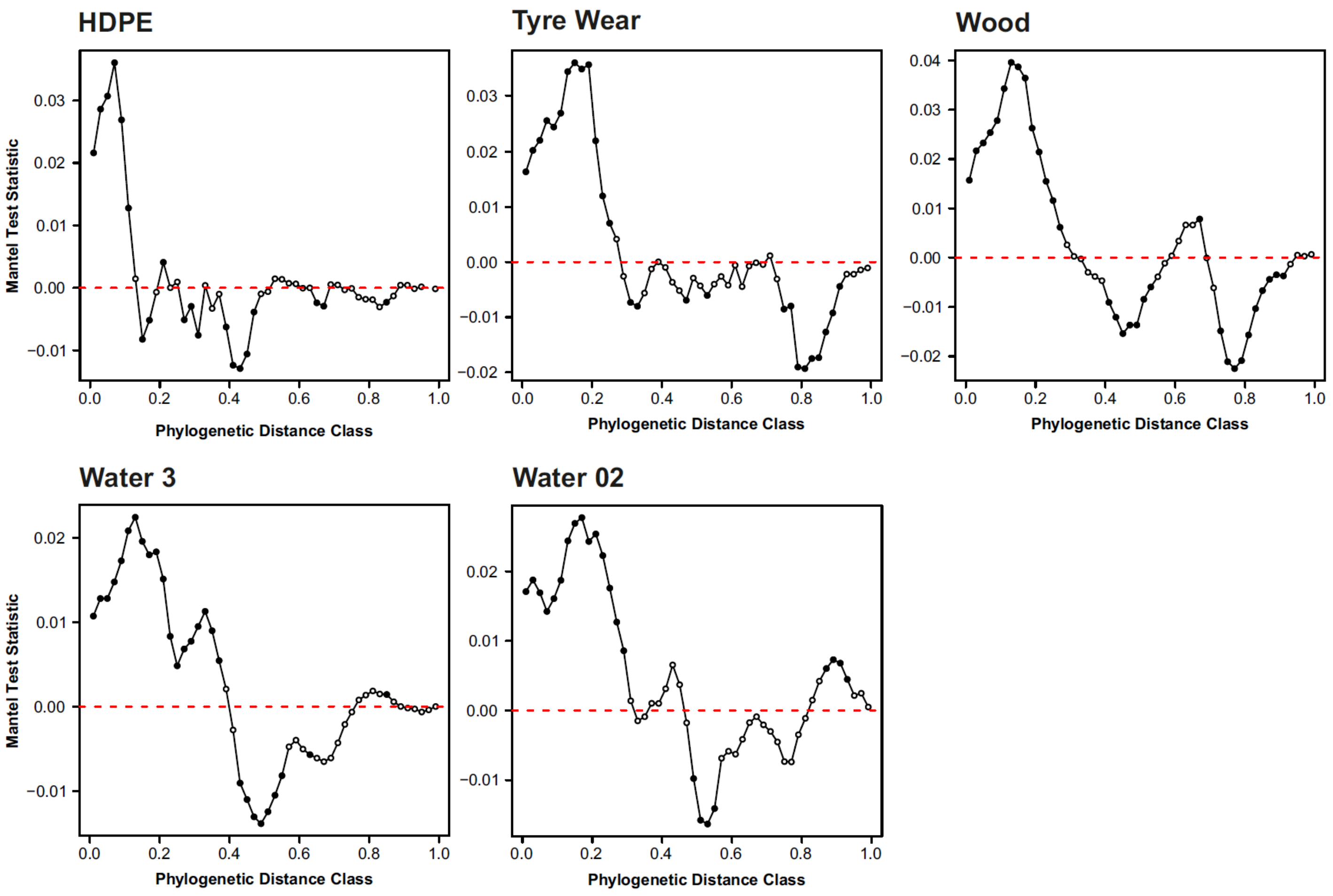

Fig. S8 Mantel correlograms displaying significant phylogenetic signals detected across short evolutionary distances for each sample type. Filled black points are indicative of a significant relationship between the most closely-related taxa and their respective ecological niches calculated based on a combination of all environmental parameters measured (Temperature, Oxygen, Salinity, Conductivity, pH). Water 3: particle-associated waterborne communities; Water 02: free-living waterborne communities.

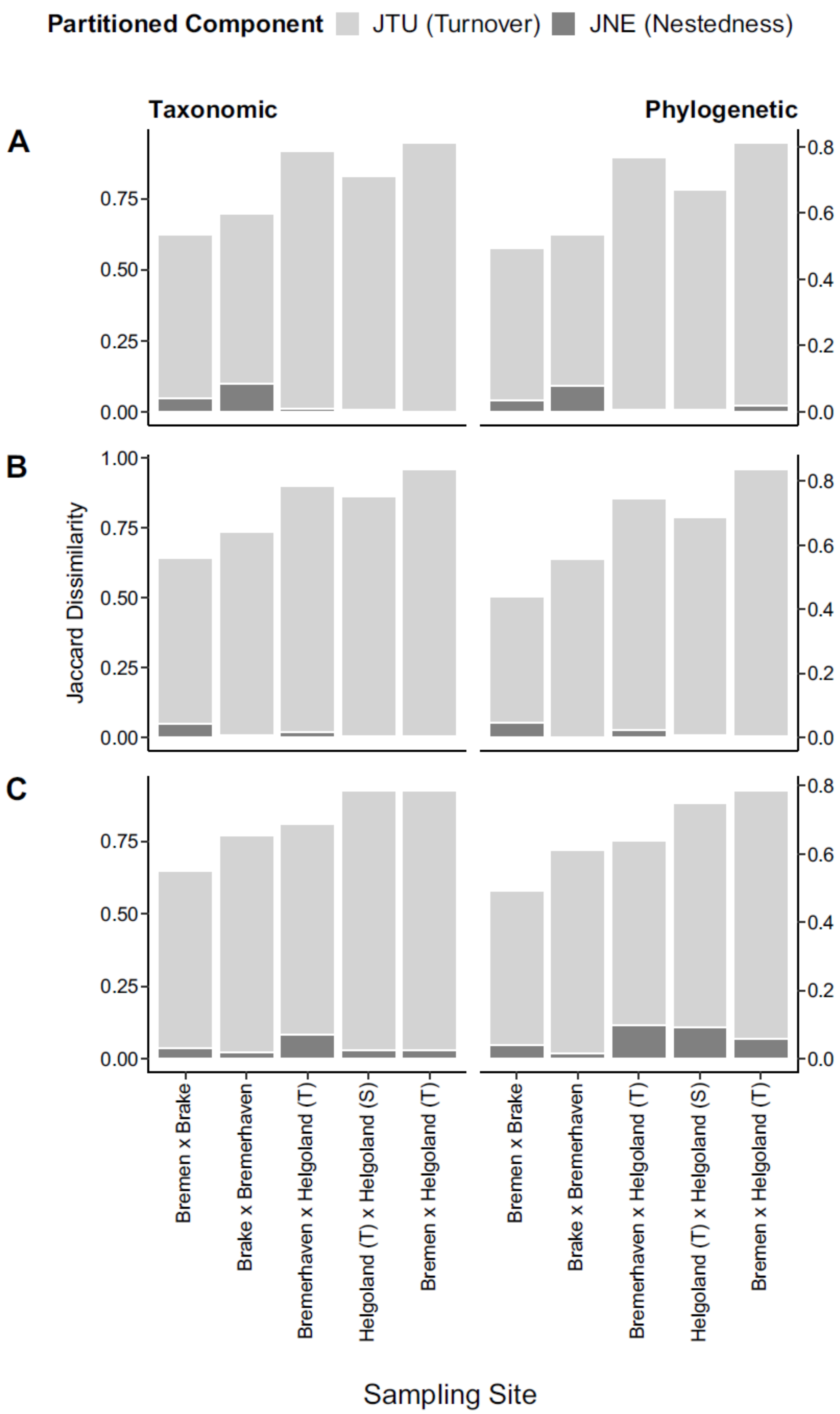

Fig. S9 Stacked bar plots showing the dissimilarities within A) HDPE, B) Tyre Wear, and C) Wood communities observed between sites, partitioned into components of turnover (JTU) and nestedness (JNE) based on Jaccard dissimilarity.

Supplementary Tables

Table S1 Information on incubation sites, and samples collected and processed in this study. (A) Descriptions of the location and conditions at each site are presented along with sampling dates and times. A total of 99 samples (45 particle-associated; P01 – P45, 54 waterborne; W01 - W54) were analysed. (B) The number of particles or volume of water from which DNA was extracted, volumes of DNA sequenced, and their respective concentrations are provided for each sample. The table is appended as an Excel document.

Table S2 Mean relative abundances (%) of bacterial phyla detected per sample type. Helgoland (T) and (S) represent samples from the transferred and stationary cages, respectively. For particle-associated (Water 3) and free living (Water 02) waterborne communities, Helgoland (T) and (S) refer to surface waters sampled from the final offshore site before and after the incubation of the transferred and stationary cages, respectively, which were pooled together. The table is appended as an Excel document.

Table S3 Mean relative abundances (%) of bacterial classes detected per sample type. Helgoland (T) and (S) represent samples from the transferred and stationary cages, respectively. For particle-associated (Water 3) and free living (Water 02) waterborne communities, Helgoland (T) and (S) refer to surface waters sampled from the final offshore site before and after the incubation of the transferred and stationary cages, respectively, which were pooled together. The table is appended as an Excel document.

Table S4 Results of the Kruskal-Wallis Rank Sums test and pairwise Dunn's Test comparing the species richness of the different sample types based on the Shannon diversity index. All *p*-values were adjusted using Benjamini-Hochberg correction ($p_{adj.}$). Significant values ($p < 0.05$) are highlighted in bold. *TW: Tyre Wear; Water 3: particle-associated waterborne communities; Water 02: free-living waterborne communities.*

		**Stationary cage**					**Transfer cage**				
		**HDPE**	**TW**	**Wood**	**Water 3**	**Water 02**	**HDPE**	**TW**	**Wood**	**Water 3**	**Water 02**
**Stationary cage**	**HDPE**										
	**TW**	**0.009**									
	**Wood**	0.235	0.237								
	**Water 3**	0.921	**0.011**	0.254							
	**Water 02**	**0.006**	0.896	0.181	**0.008**						
**Transfer cage**	**HDPE**	0.711	**0.000**	**0.044**	0.647	**0.000**					
	**TW**	**0.004**	0.693	0.246	**0.006**	0.576	**0.000**				
	**Wood**	0.334	**0.020**	0.569	0.385	**0.014**	**0.025**	**0.004**			
	**Water 3**	0.530	**0.006**	0.330	0.568	**0.004**	0.058	**0.000**	0.560		
	**Water 02**	**0.015**	0.314	0.550	**0.021**	0.239	**0.000**	0.288	**0.020**	**0.000**	

$\chi^2 = 72.540$; df: 9; $p =$ **4.826E-12**

Table S5 Results of the Kruskal-Wallis Rank Sums test and pairwise Dunn's Test comparing the species richness of the different sample types based on the Gini-Simpson diversity index**.** All *p*-values were adjusted using Benjamini-Hochberg correction ($p_{adj.}$). Significant values ($p < 0.05$) are highlighted in bold. *TW: Tyre Wear; Water 3: particle-associated waterborne communities; Water 02: free-living waterborne communities.*

		**Stationary cage**					**Transfer cage**				
		**HDPE**	**TW**	**Wood**	**Water 3**	**Water 02**	**HDPE**	**TW**	**Wood**	**Water 3**	**Water 02**
**Stationary cage**	**HDPE**										
	**TW**	**0.012**									
	**Wood**	0.396	0.117								
	**Water 3**	0.861	**0.018**	0.505							
	**Water 02**	**0.037**	0.663	0.299	0.056						
**Transfer cage**	**HDPE**	0.603	**0.000**	0.070	0.479	**0.001**					
	**TW**	**0.007**	0.645	0.138	**0.013**	0.921	**0.000**				
	**Wood**	0.477	**0.016**	0.659	0.601	0.063	**0.033**	**0.002**			
	**Water 3**	0.661	**0.003**	0.480	0.835	**0.019**	0.071	**0.000**	0.527		
	**Water 02**	**0.036**	0.203	0.491	0.065	0.525	**0.000**	0.185	**0.033**	**0.000**	

χ^2 = 70.330; df: 9; *p* = **1.314E-11**

Table S6 Results of the Kruskal-Wallis Rank Sums test and pairwise Dunn's Test comparing the phylogenetic diversity of the different sample types based on Faith's PD. All *p*-values were adjusted using Benjamini-Hochberg correction ($p_{adj.}$). Significant values ($p < 0.05$) are highlighted in bold. *TW: Tyre Wear; Water 3: particle-associated waterborne communities; Water 02: free-living waterborne communities.*

		Stationary cage					Transfer cage				
		**HDPE**	**TW**	**Wood**	**Water 3**	**Water 02**	**HDPE**	**TW**	**Wood**	**Water 3**	**Water 02**
**Stationary cage**	**HDPE**										
	**TW**	0.228									
	**Wood**	0.092	0.646								
	**Water 3**	0.832	0.332	0.145							
	**Water 02**	**0.009**	0.183	0.397	**0.019**						
**Transfer cage**	**HDPE**	0.794	0.072	**0.014**	0.610	**0.000**					
	**TW**	**0.044**	0.676	0.902	0.087	0.226	**0.000**				
	**Wood**	0.344	0.618	0.259	0.529	**0.023**	**0.043**	0.100			
	**Water 3**	0.604	0.319	0.093	0.803	**0.004**	0.112	**0.007**	0.460		
	**Water 02**	**0.039**	0.661	0.887	0.075	0.185	**0.000**	0.951	0.071	**0.001**	

χ^2 = 52.927; df: 9; *p* = **3.018E-08**

Table S7 Results of the Kruskal-Wallis Rank Sums test and pairwise Dunn's Test comparing the mean phylogenetic relatedness computed for the different sample types based on the nearest taxon index (NTI). All *p*-values were adjusted using Benjamini-Hochberg correction ($p_{adj.}$). Significant values ($p < 0.05$) are highlighted in bold. *TW: Tyre Wear; Water 3: particle-associated waterborne communities; Water 02: free-living waterborne communities.*

		**Stationary cage**					**Transfer cage**				
		**HDPE**	**TW**	**Wood**	**Water 3**	**Water 02**	**HDPE**	**TW**	**Wood**	**Water 3**	**Water 02**
**Stationary cage**	**HDPE**										
	**TW**	0.085									
	**Wood**	0.538	0.267								
	**Water 3**	0.113	0.879	0.374							
	**Water 02**	0.422	0.386	0.864	0.465						
**Transfer cage**	**HDPE**	0.508	0.119	0.901	0.189	0.757					
	**TW**	0.119	0.553	0.461	0.734	0.603	0.147				
	**Wood**	0.414	0.183	0.968	0.267	0.882	0.830	0.267			
	**Water 3**	**0.003**	0.788	0.079	0.610	0.120	**0.000**	0.094	**0.001**		
	**Water 02**	**0.027**	0.902	0.203	0.979	0.325	**0.008**	0.451	**0.024**	0.268	

$\chi^2 = 42.503$; df: 9; $p =$ **2.659E-06**

Table S8 Results of a post hoc two-way PERMANOVA and PERMDISP comparing the different sample types collected from the transferred cage and surrounding waters at each sampling site based on taxonomic and phylogenetic dissimilarity metrics. All *p*-values were adjusted using Benjamini-Hochberg correction ($p_{adj.}$). Significance ($p < 0.05$) is highlighted in bold and additionally starred when corresponding pairwise PERMDISP *p*-values were also significant (*). *TW: Tyre Wear; Water 3: particle-associated waterborne communities; Water 02: free-living waterborne communities; t: pseudo t-statistic.* The table is provided as an Excel document.

Table S9 Results of a post hoc two-way PERMANOVA and PERMDISP comparing between the different sampling sites for each sample type collected from the transferred cage and surrounding waters based on taxonomic and phylogenetic dissimilarity metrics. All *p*-values were adjusted using Benjamini-Hochberg correction ($p_{adj.}$). Significance ($p < 0.05$) is highlighted in bold and additionally starred when corresponding pairwise PERMDISP *p*-values were also significant (*). *TW: Tyre Wear; Water 3: particle-associated waterborne communities; Water 02: free-living waterborne communities; t: pseudo t-statistic.* The table is provided as an Excel document.

Table S10 ßNTI and RCBray matrices for (A) HDPE, (B) Tyre Wear, (C) Wood, (D) Water 3, and (E) Water 02 communities across sites.

Table S11 Similarity of Percentages (SIMPER) analysis showing bacterial taxa with a contribution > 1% to dissimilarities within (A) HDPE, (B) Tyre Wear, (C) Wood, and (D, E) waterborne communities between sites. The sum (∑) and mean (μ) percentage contribution of each bacterial family to differences observed between sites and their average dissimilarities (%) are shown. The number of ASVs (N_{ASV}) belonging to each family, their total and mean abundance at each site, and the difference in the mean counts (δ) detected between each pair of sites are also presented. Helgoland (T) and (S) represent samples from the transferred and stationary cages, respectively. For particle-associated (Water 3) and free-living (Water 02) waterborne communities, Helgoland (T) refers to surface waters sampled from the final offshore site before and after the incubation of the transferred cage which were pooled together. The table is provided as an Excel document.

Table S12 Results of a one-way PERMANOVA showing the effect of Sample Type on community structure differences between samples from the transferred and stationary cages based on taxonomic and phylogenetic dissimilarity metrics. *p*-values were adjusted using Benjamini-Hochberg correction ($p_{adj.}$) and significance ($p < 0.05$) highlighted in bold. *d.f.: degrees of freedom; SS: sum of squares; Sq. root: square root.*

**Resemblance**	**Sources of variation**	**d.f.**	**SS**	***pseudo F***	$p_{adj.}$	**Sq. root**
Bray Curtis	Sample Type	7	93443	8.046	**0.0001**	52
	Residuals	28	46453			41
	Total	35	139900			
Jaccard	Sample Type	7	77600	4.203	**0.0001**	44
	Residuals	28	73850			51
	Total	35	151450			
weighted UniFrac	Sample Type	7	1.151	7.681	**0.0001**	0.183
	Residuals	28	0.600			0.146
	Total	35	1.751			
unweighted UniFrac	Sample Type	7	6.411	5.865	**0.0001**	0.421
	Residuals	28	4.373			0.395
	Total	35	10.784			

Table S13 Results of a post hoc one-way PERMANOVA and PERMDISP comparing between samples collected from the transferred and stationary cages at the final offshore site (Helgoland only) based on taxonomic and phylogenetic dissimilarity metrics. All *p*-values were adjusted using Benjamini-Hochberg correction ($p_{adj.}$). Significance ($p < 0.05$) is highlighted in bold and additionally starred when corresponding pairwise PERMDISP p-values were also significant (*). (T) and (S) represent samples from the transferred and stationary cages, respectively. The table is provided as an Excel document.
